# Small molecule drug and biotech drug interaction prediction based on multi-modal representation learning

**DOI:** 10.1186/s12859-022-05101-2

**Published:** 2022-12-27

**Authors:** Dingkai Huang, Hongjian He, Jiaming Ouyang, Chang Zhao, Xin Dong, Jiang Xie

**Affiliations:** 1 School of Computer Engineering and Science, Shanghai University, Shanghai, 200444 China; 2 School of Medicine, Shanghai University, Shanghai, 200444 China

**Keywords:** Drug–drug interactions, Multi-modal representation learning, PU-sampling

## Abstract

**Background:**

Drug–drug interactions (DDIs) occur when two or more drugs are taken simultaneously or successively. Early detection of adverse drug interactions can be essential in preventing medical errors and reducing healthcare costs. Many computational methods already predict interactions between small molecule drugs (SMDs). As the number of biotechnology drugs (BioDs) increases, so makes the threat of interactions between SMDs and BioDs. However, few computational methods are available to predict their interactions.

**Results:**

Considering the structural specificity and relational complexity of SMDs and BioDs, a novel multi-modal representation learning method called Multi-SBI is proposed to predict their interactions. First, multi-modal features are used to adequately represent the heterogeneous structure and complex relationships of SMDs and BioDs. Second, an undersampling method based on Positive-unlabeled learning (PU-sampling) is introduced to obtain negative samples with high confidence from the unlabeled data set. Finally, both learned representations of SMD and BioD are fed into DNN classifiers to predict their interaction events. In addition, we also conduct a retrospective analysis.

**Conclusions:**

Our proposed multi-modal representation learning method can extract drug features more comprehensively in heterogeneous drugs. In addition, PU-sampling can effectively reduce the noise in the sampling procedure. Our proposed method significantly outperforms other state-of-the-art drug interaction prediction methods. In a retrospective analysis of DrugBank 5.1.0, 14 out of the 20 predictions with the highest confidence were validated in the latest version of DrugBank 5.1.8, demonstrating that Multi-SBI is a valuable tool for predicting new drug interactions through effectively extracting and learning heterogeneous drug features.

## Introduction

DDIs refer to the phenomenon in which one drug alters the pharmacological effects of another drug when two or more drugs are taken simultaneously or sequentially [1]. DDIs may lead to unexpected adverse drug side effects [2]. Early detection of DDIs can effectively prevent medical errors and reduce healthcare costs. Early on, researchers identified DDIs by wet experiments and later used high-throughput screening and in vivo models. However, these methods are time-consuming and labor-intensive, so systematic combinatorial screening of potential DDIs remains challenging. To reduce the cost in time and money, computational methods are gaining more highlights. Early researchers collected drug data from the literature, reports, etc., to predict DDIs, and some proposed machine learning methods to predict DDIs [3].

The current DDI prediction methods based on machine learning are broadly classified into similarity-based and network-based methods. Similarity-based methods assume that drugs with similar properties interact with the same drugs [4]. Early research used molecular structure similarity information to identify new DDI [4]. Since single molecular structure information is insufficient to express drug characteristics, [5] established a DDI prediction model by integrating multiple drug similarity measures. Moreover, four classifiers were adopted to construct predictive models simultaneously [6]. With the advancement of deep learning research, DeepDDI [7] used the drug name and chemical structure as inputs to the deep neural network (DNN) to predict the DDI types of drug pairs and drug-food component pairs. The DDIMDL [8] constructed four sub-models using features of each drug and used joint deep learning DNNs to predict DDI-related events. The latest study combines two drugs in four different ways. It feeds the combined drug feature representation into four different drug fusion networks to obtain the latent feature vectors of the drug pairs [9]. The network-based method converts the graph into a low-dimensional space that preserves the information of the structural graph and then uses the learned low-dimensional representation as a feature for prediction. [10] constructed a network based on chemical structure and side effect similarities of drugs and applied a label propagation algorithm to identify DDIs. Decagon, a graph convolutional neural network, was designed for running on large multi-modal graphs [11]. Based on this model, a three-picture information dissemination (TIP) model improved prediction accuracy and time and space efficiency [12].

Generally, most of the state-of-the-art methods mentioned above only predict whether there exists a DDI between a pair of SMDs. As the number of biotech drugs (BioDs) increases, so makes the threat of adverse interactions between SMD and BioD. Biologics are medicines derived from living cells or biological processes [13, 14]. Unlike the relatively simple structure of SMDs, the structural complexity of biologics makes the characterization of SMD and BioD drug pairs difficult [15]. Besides that, most methods straightforwardly employ random sampling in unlabeled data for generating negative samples, resulting in many false negatives in the sampled negative samples [16, 17].

To overcome these limitations, we propose a multi-modal representation learning method called Multi-SBI for predicting the interaction between SMDs and BioDs. Considering the structural specificity and relational complexity of SMDs and BioDs, we first apply multi-modal representation learning to learn drug features thoroughly. On the one hand, it takes the one-dimensional sequence information of two types of drugs as input. It learns the sequence features separately through traditional methods such as convolutional neural networks (CNN). On the other hand, the association information of all drug nodes in the heterogeneous network is encoded as a one-dimensional feature vector. Then, we adopt the PU-sampling to select high-confidence negative samples, which can reduce sampling noise. Finally, different modal drug pair features of dimensionality-reducing are input into DNN classifiers to predict the new SMD-BioD interaction (SBI). In the SBI prediction experiment on the public data set, the fully designed Multi-SBI has a higher accuracy rate and performs better than several state-of-the-art methods. In addition, in retrospective analysis, the high-confidence SBI predicted by the Multi-SBI model has been verified by the latest version of the DrugBank database, proving that our model has solid predictive capabilities. To summarize, the main contributions of this paper are:

- A multi-modal representation learning model is developed for predicting SBI that can effectively characterize drugs through the structural information of drugs and topological associations in heterogeneous networks.
- PU-sampling is designed to extract unbalanced unlabeled negative samples, which can extract negative samples with high confidence.
- The experiments show that Multi-SBI has achieved excellent performance in all indicators (accuracy, AUC, AUPR, F1, precision, and recall). It yielded higher performance in predicting SBI.

The rest of this paper is structured as follows. The “Methods” section introduces the basic concepts and processes of Multi-SBI. In addition, the experiments are analyzed in the “Experiments” section. Next, the Multi-SBI is analyzed and verified through various experiments in the “Discussion” section, finally showing the retrospective analysis. In the “Conclusion” section, the work that has been carried out and the direction of future research are summarized.

## Methods

### Problem description

**Fig. 1:**
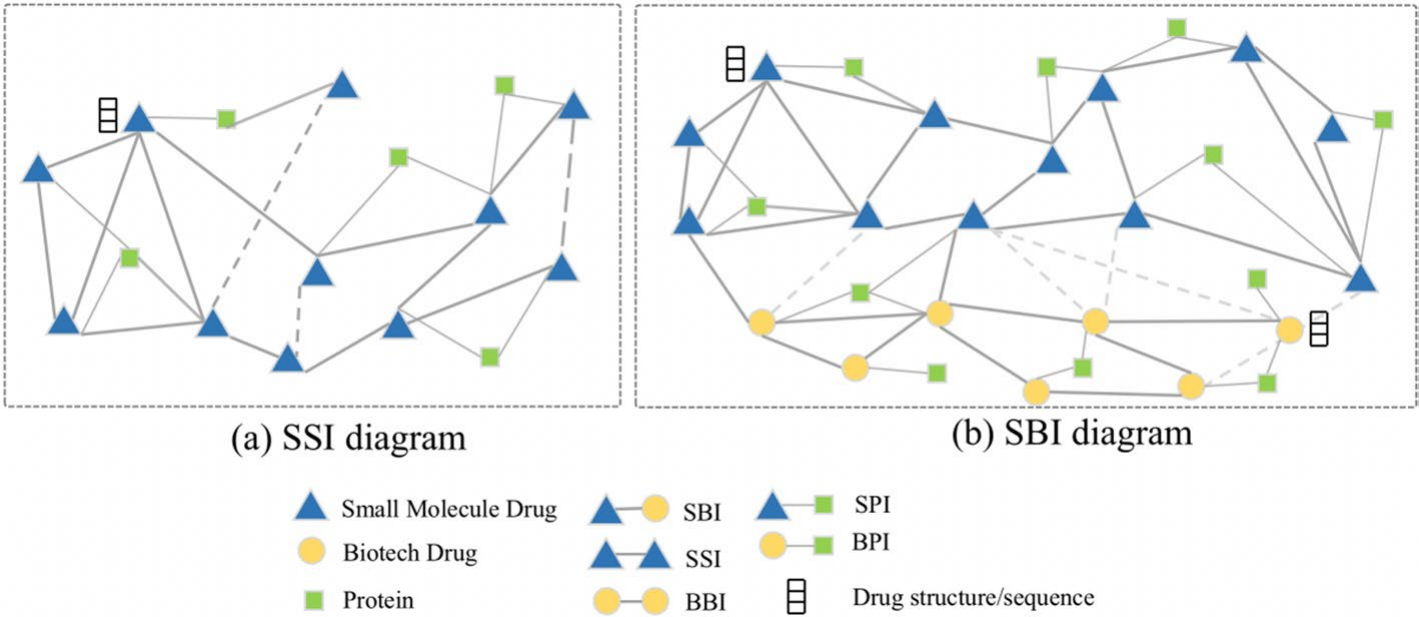
Two DDI diagrams. **a** The traditional drug interaction (SSI) prediction task contains one type of drug node and two types of node associations. **b** Two types of drug nodes and five types of node associations are included in the SMD-BioD interaction (SBI) prediction task

As shown in Fig. 1a, conventional DDI prediction focuses on SMDs, only containing one type of drug node and drug-protein association, and drug features only consist of structural forms like SMILES. In comparison, in Fig. 1b after adding BioDs three types of nodes and five types of associations make the SBI prediction more complex. Furthermore, BioDs are composed of amino acid sequences, which differ from SMDs. The other problem is that there are no accurately annotated negative samples in the database, which means the prediction results depend on the sampling strategy. To solve the above problem, we use multi-modal representation learning to learn complex drug pair features and apply the PU-sampling method to deal with imbalanced data.

### Multi-modal representation learning

The performance of deep learning methods is largely reflected in efficient data representation, which means that a model can automatically discover the representation needed for feature extraction or classification from raw data using a set of techniques. This process is called representation learning, which is one of the fundamental steps in end-to-end deep learning. Many works have integrated deep learning methods into the feature representation design of input data to more easily extract useful feature information [18–24].

**Fig. 2:**
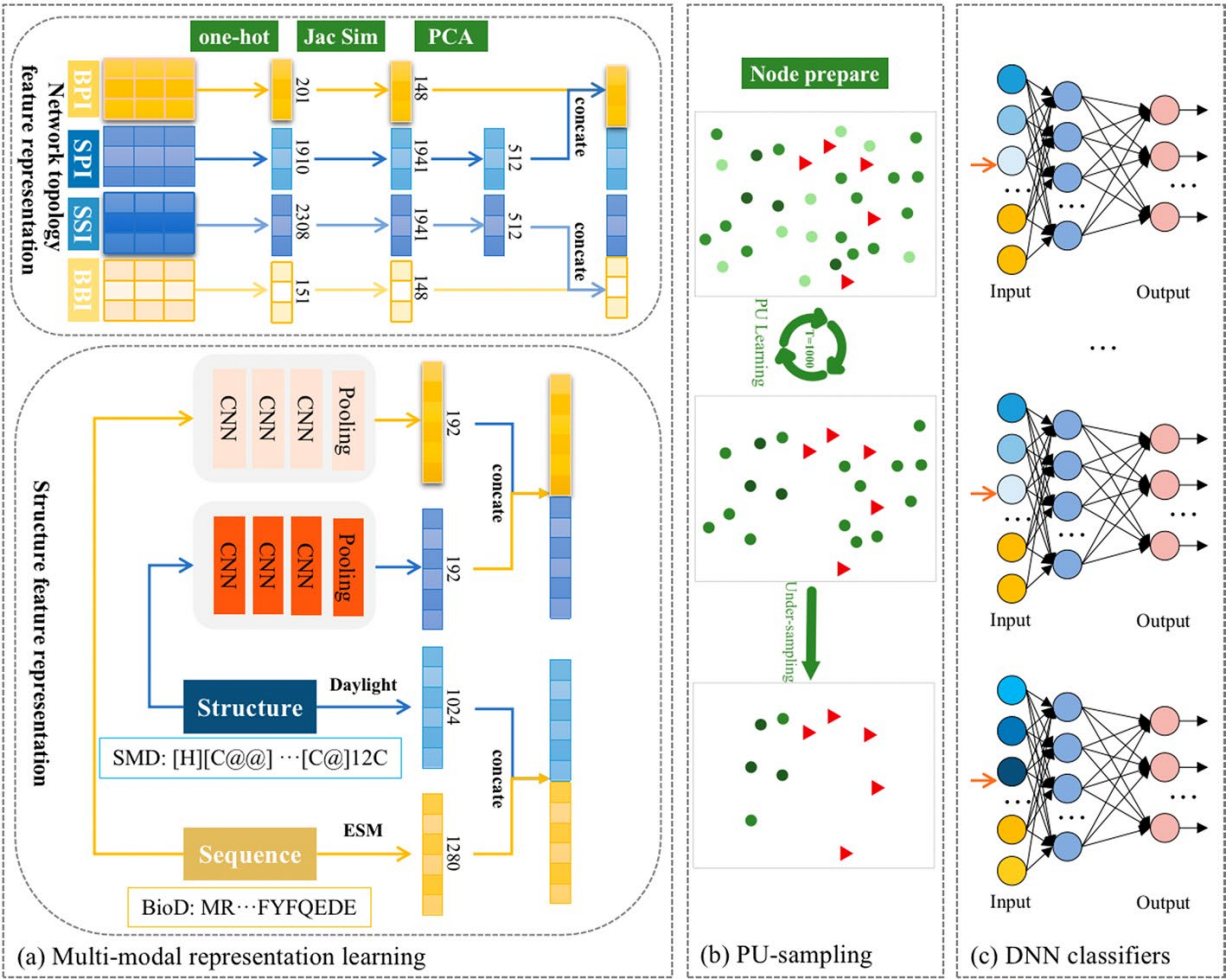
The overall workflow of Multi-SBI. **a** Multi-modal representation learning obtains structure and network topology features from the diverse drug types. **b** PU-sampling is introduced to obtain negative samples with high confidence from the unlabeled data set. **c** Combining multi-modal data into the DNN classifiers provides a complementary view of SBI

The workflow of Multi-SBI is depicted in Fig. 2. Considering the structural specificity and relational complexity of SMD and BioD, our multi-modal representation learning comprises two separate pathways. As shown in Fig. 2a, structure feature representation and network topology representation are obtained. In addition to traditional methods, we propose two independent three-layer 1D-CNN blocks to learn the drug structure features from the sequence input(Structure/Sequence). After one-hot encoding the four interconnected networks (SMD-protein interaction (SPI), BioD-protein interaction (BPI), SMD-SMD interaction (SSI), and BioD-BioD interaction (BBI)), the similarity is encoded into a heterogeneous network to fully characterize drugs relational topology representation.

### Structure feature representation

In previous studies, the information about the chemical structure of SMD derives from the drug's chemical substructure, i.e., molecular fingerprints. Here, we apply Chemistry Development Kit (CDK) [25], an open-source tool commonly used in DDI prediction, to generate substructures. In more detail, we select the daylight fingerprint method in the CDK toolkit, which is the most typical representative of the topological molecular fingerprint. The raw inputs are the simplified molecular input line entry system (SMILES) of all drugs downloaded from DrugBank [26], and 1024-dimensional molecular structure features of SMDs are extracted after the algorithm.

The structure of BioD is similar to protein, both of which are composed of primary amino acid sequences. Many feature extraction methods are based on amino acid sequences [27, 28]. Expressly, these features usually represent information about the physicochemical properties or positions of amino acids that appear in the protein sequence. However, BioD sequence data are scarce in the field of a drug interaction. This study has only 148 unique BioDs, and traditional methods cannot extract highly discriminative features in such a small amount of data. Therefore, here we utilize ESM [29] to pre-train BioDs. Because the ESM specially adopts a masking language to model the target and contains information that is not available in other feature extraction methods. Given a BioD, we intercept the top 1024 bits of its amino acid sequence and encode it through the ESM algorithm. In this way, each BioD is encoded into a 1280-dimensional vector.

Traditional methods directly apply molecular fingerprints or molecular descriptors of drugs and targets without considering the local connection between atoms and the chemical structure of amino acids [30, 31]. In addition to daylight and ESM, we integrate two 1D-CNN blocks for the original sequence features to complementarily extract the complex chemical information and contextual relationships between the local structures in the sequence.

In this study, the SMILES string for SMD consists of 64 different characters, and BioD consists of 25 different characters. We represent each character with the corresponding integer (e.g. "[": 1, "H": 2, "@": 3). In addition, both SMILES and amino acid sequences have different lengths in order to represent the two classes of drugs efficiently, we convert each SMILES and amino acid sequence into embedding vectors of length 1000 and 100, and input them into a two-channel CNN in the module.

**Fig. 3:**
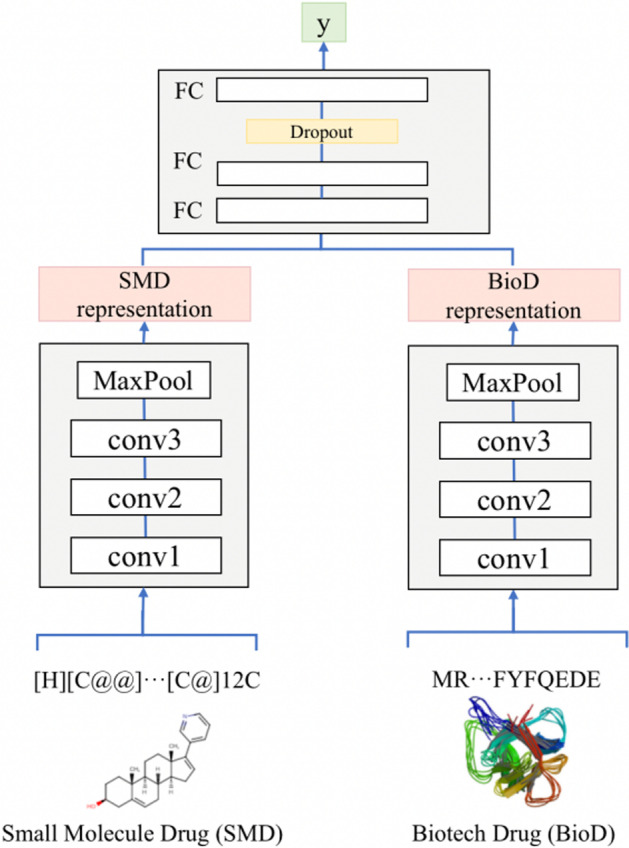
Two independent three-layer 1D-CNN blocks extract context structure information from different drug sequence inputs. The length of the convolution filters is fixed to 8, while the filter numbers are 64, 128, and 192, respectively

As shown in Fig. 3, the two-channel CNN module in this study contains two independent CNN blocks, and each aims at learning representations from SMILES strings and amino acid sequences. For each CNN block, we use three consecutive 1D convolutional layers with an increasing number of filters. The second layer has twice as many filters as the first layer, and the third convolutional layer has three times as many filters as the first. The last layer is the maximum pooling layer. The output of the maximum pooling layer are connected and fed into the three-layer DNN classifier.

### Network topology feature representation

The integration of bioinformatics prior knowledge can effectively improve the accuracy of prediction [8]. Therefore, in addition to applicable drug structure and sequence features, we use four network topology features from the DrugBank database as another modality.

The topology network inputs for Multi-SBI are constructed based on known prior knowledge: SSI, BBI, SPI, and BPI. Among them, the protein in the SPI and BPI includes four parts: target, enzyme, carrier, and transporter. Multi-SBI first performs one-hot encoding on each network to obtain the distribution of each drug node, which captures its topological relationship to all other nodes in the heterogeneous network. We generate a 2308-dimensional SSI embedding and a 1910-dimensional SPI embedding for each SMD through the one-hot encoding strategy. The value (1 or 0) indicates the presence or absence of the protein-related interaction with the corresponding drug. Similarly, we generate the 151-dimensional BBI embedding and the 201-dimensional BPI embedding for BioDs.

A critical problem of direct one-hot encoding is that the calculated topological relationship is not entirely accurate, partly because of the noisy, incomplete, and high-dimensional nature of biological data. To speed up the prediction process and eliminate noise as much as possible, we compress features to reduce sparsity. Instead of using bit vectors, we use the Jaccard similarity metric to calculate paired drug–drug similarity from bit vectors. Jaccard similarity is calculated by Eq. (1):

1 $$J\left( {A,B} \right) = \frac{| A \cap B |}{{\left| A \right| + \left| B \right| - \left| {A \cap B} \right|}}$$

Among them, A and B are the set forms of the position vectors of the two drugs; |A ∩ B| is the intersection of A and B. Using Jaccard similarity, we convert topological features of SMD drugs and BioD drugs to 1941 and 148 dimensions (determined by the number of drugs). Because SMD drugs have 1941 dimensions, we use PCA to reduce the feature dimension to 512 dimensions.

Finally, we obtain the drug pair feature consisting of two types of sequence features and two types of topological features.

### PU-sampling

In some applications, such as drug interaction prediction, only positive cases are known and labeled, while unlabeled data may include negative and unlabeled positive cases. Previous methods used experimentally verified DDI as positive samples and randomly generated negative samples to learn predictive models. However, randomly generated negative samples may include unknown true positive samples. A classifier trained with such randomly generated negative samples may produce high cross-validation accuracy, but it is likely to perform poorly on independent real test data set. Therefore, screening highly reliable negative samples is essential to improve the effectiveness of computational prediction methods [32].

**Fig. 4:**
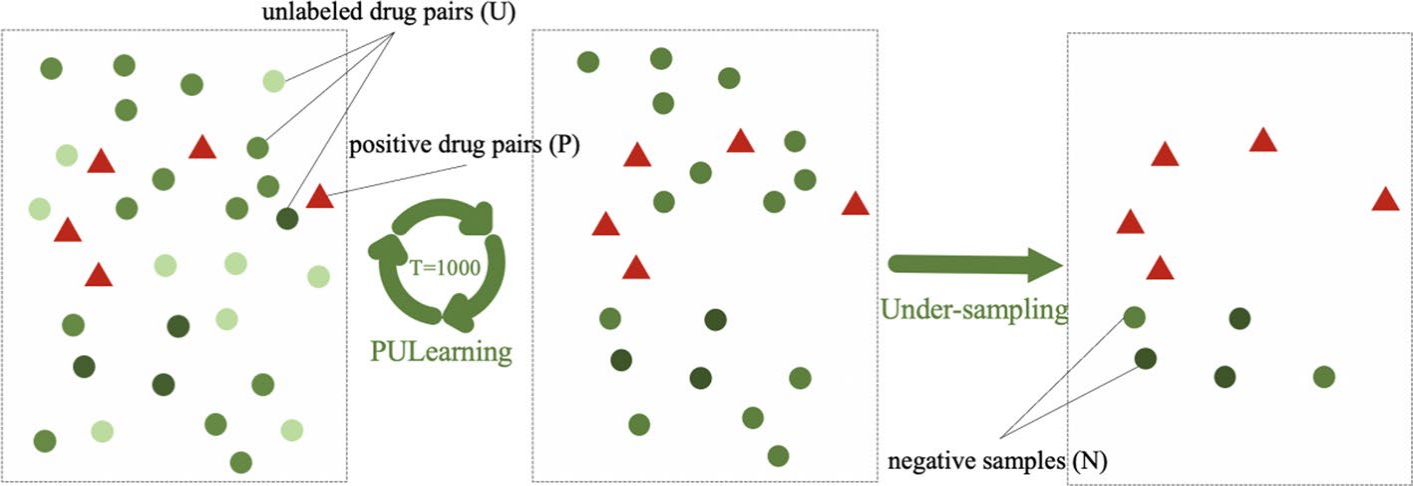
PU-sampling flow chart

As shown in Fig. 2b, to address the unbalanced data set problem in DDI prediction, we introduce an undersampling method, PU-sampling, based on Positive-unlabeled learning (PU Learning) [33]. The core concept of PU Learning is converting positive and unlabeled examples into a series of supervised binary classification problems discriminating the known positive examples from random subsamples of the unlabeled set. As more details are shown in Fig. 4, positive samples are labeled with red triangles. Firstly, PU-sampling scores all unlabeled examples through many simple decision tree classifiers. Then removes low-confidence negative sample drug pairs that are painted in light green circles. Finally, during the training process, high confidence samples are selected from the remaining unlabeled set with the same number of positives to compose the 1:1 balanced data set. As will be introduced in the “Experiment” section, there are 148 BioDs and 1,941 SMDs in the data set, generating 287,268 potential SBI drug pairs. However, only 40,959 SBI are verified positive in DrugBank. The remaining 246,309 are unlabeled. Here, we denote positive drug pairs as set P, unlabeled drug pairs as set U, and selected high-confidence negative drug pairs as N, correspondingly. The PU-sampling algorithm is as follows:

1. Randomly select the same number of P from U temporarily considered as negative in binary classification, and utilize the decision tree model to evaluate the unlabeled examples with a score from 0(negative) to 1(positive);
2. Repeat step (1) T times and record the scores from the classifiers, which means T decision tree models have been trained and the unlabeled drugs have been evaluated many times. It is believed that the average score can be used as the confidence of the negative samples;
3. Finally, after sorting all the scores, set 1 as the threshold to eliminate positive samples. Then samples with a score close to 0 can be regarded as high-confidence negative. Because the "true" negative samples theoretically are distinguishable from the labeled positive drugs, whose values should be very close to zero. Thus samples with the lowest score are taken as the negative samples set N in the following experiments.

Finally, as the positive samples are 40,959, the same number of negative samples were retained from 246,309 unlabeled drug pairs.

### DNN construction

Multi-SBI is designed as a multi-classification model that can predict multiple SBI types for a given drug pair (multiple output neurons are activated simultaneously, and each neuron represents one SBI type). In this work, we adopt "DNN" as the multivariate classifier. Since there are four types of feature, we construct four sub-models based on each type of feature using the DNN. The average operator combines the outputs from sub-models to produce the final prediction.

Figure 2c shows that each prediction sub-model concatenates a pair of SMD and BioD embedding vectors, which is input to the fully connected layer to calculate the interacting probability. The output layer has 49 output neurons, representing the 49 classification types considered in this study. These output neurons have activity values between 0 (no interaction) and 1 (possible interaction), which can be considered a probability [34].

As shown in Fig. 2c, the DNN consists of three layers, with the number of nodes being 512, 256, and 49.

## Experiments

### Data resources

The number of drugs in the database has dramatically increased in the past few years. The DrugBank [35] database integrates bioinformatics and chemoinformatics resources, providing detailed drug data. We collect features about SBI and drugs from DrugBank 5.1.8 released in January 2021: molecular structure of SMD, amino acid sequence of BioD, SMD-SMD interaction (SSI), BioD-BioD (BBI) interaction, SMD-Protein Interaction (SPI), BioD-Protein Interaction (BPI) and known SBI. We select drugs with at least one SBI and SPI, and the experimental data obtained are shown in Table 1.

**Table 1:** Data statistics from DrugBank

	Data Category	Number
Entity	SMD	1941
	BioD	148
	Protein	1910
Interaction	(SMD-SMD)SSI	2308
	(BioD-BioD)BBI	151
	(SMD-Protein)SPI	1910
	(BioD-Protein)BPI	201
	(SMD-BioD)SBI	40959

For SBI classification categories, we use a similar method in [8] to extract SBI and define the expression of SBIs as a quaternary structure: (drug A, drug B, mechanism, action). The "mechanism" means the effect of drugs in terms of metabolism, serum concentration, therapeutic efficacy, and other aspects. The "action" means an increase or decrease of the corresponding mechanism. With the above definition, we obtain 48 events to describe the existing SBI types. When it is worth noting that in order to facilitate analysis [8], deleted the DDI related to a single event and selected events with more than 10 DDIs. Although such label preprocessing is beneficial to program design and improves the accuracy of drug interaction prediction, it is unreasonable in actual clinical trials. Therefore, to retain all DDIs and perform cross-validation, we reserved events with no more than 10 DDIs into a single category to facilitate subsequent experiments.

**Fig. 5:**
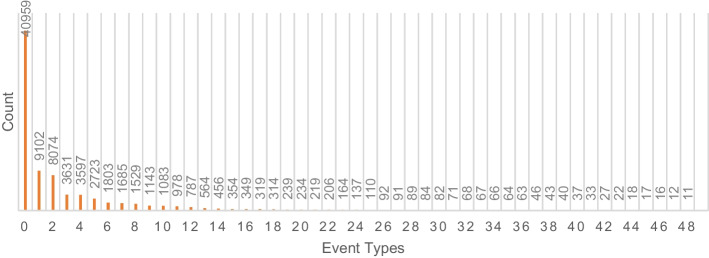
All classification categories (category 0 for negative samples and 1 to 48 for SBI types)

The number of 48 different SBI events and negative samples (as category 0) is described in Fig. 5. Due to the unbalanced data distribution, the negative and most positive samples are centralized on the left side of the histogram.

### Evaluation metrics

We evaluate the prediction performance of Multi-SBI using a five-fold cross-validation procedure, in which 80% of the drug pairs are randomly selected as the training set, and the remaining 20% of the drug pairs are used as the test set. The final performance of the model takes the average of the five-fold results. For each fold of each prediction model, the following indicators are calculated:

2 $$ACC=\frac{TP+TN}{TP+FP+TN+FN}$$

3 $$AUC=\sum_{i=1}^{n}{TPR}_{i}\Delta {FPR}_{i}$$

4 $$AUPR=\sum_{j=1}^{n}{Pre}_{j}\Delta {Rec}_{j}$$

5 $$F1=\frac{2*Sen*Pre}{Sen+Pre}$$

6 $$Pre=\frac{TP}{TP+FP}$$

7 $$Rec=TPR=\frac{TP}{TP+FN}$$

8 $$FPR=\frac{FP}{FP+TN}$$

where TP means true positive, TN means true negative, FP means false positive, FN means false negative, $i$ is $ith$ true-positive/false-positive operating point, and $j$ is $jth$ precision/recall operating point.

### Experimental setup

There are four essential hyper-parameters in our model, namely the layer number, optimizer, learning rate, and dropout rate on the model.

First, we discuss the number of DNN layers. We set a rule that the number of neurons in a layer is half the previous layer and then fixed the number of neurons in the last hidden layer to 256. We consider 2, 3, 4, and 5 hidden layers and adopt a three-layer structure (the number of nodes is 512, 256, and 49, respectively) because it can achieve the best performance.

In order to optimize the model, we use the Adam optimizer [36] to train up to 100 epochs (training iterations) with a learning rate of 0.3 and stop training if the verification loss does not decrease in 10 epochs [37]. This strategy can prevent over-fitting while considerably speeding up the training process.

In order to make the model generalize well to the unobserved drug pairs, we apply regular dropout [38] to hidden layer units. We set the dropout rate from 0 to 0.5 in steps of 0.1 and get the highest Accuracy (ACC) when dropout is equal to 0.3.

### Feature evaluation

Here, we first evaluate the impact of multi-modal features on model performance. While keeping other parameters constant, we use different drug features for drug representation. Specifically, four types of features: CNN, daylight/EMS, SPI/BPI, and SSI/BBI are used to compare. Then we test the following 15 drug feature combinations to make predictions.

It can be seen in Table 2, using only CNN, that the performance indicators of the model are significantly higher than other single features. The results show that CNN can more effectively represent long-distance associations and global information in long sequences, thereby improving the performance of predicting SBI. The performance of the feature combination of daylight/EMS and CNN is higher than that of daylight/EMS or CNN alone, which indicates that the combination of different feature representations of the same data source can extract features from different perspectives and thus improve prediction accuracy. In addition, the best results can be obtained when all modalities are used, proving the superiority of our proposed multi-modal representation learning framework, combing drug structure information and the relevant information of heterogeneous networks. Therefore, we choose CNN + daylight/EMS + SPI/BPI + SSI/BBI as the model feature.

**Table 2:** The performance of Multi-SBI with different feature combinations

Method	ACC	AUC	AUPR	F1	Pre	Rec
CNN	0.9336	0.9993	0.9794	0.8016	0.8111	0.8221
daylight/EMS	0.9106	0.9991	0.9652	0.8047	0.8683	0.7983
SPI/BPI	0.7736	0.9960	0.8770	0.4772	0.5741	0.4577
SSI/BBI	0.8211	0.9976	0.9190	0.5623	0.6503	0.5219
CNN + daylight/EMS	0.9427	0.9995	0.9807	0.8337	0.8569	0.8410
CNN + SPI/BPI	0.9353	0.9992	0.9705	0.8005	0.8302	0.8028
CNN + SSI/BBI	0.9381	0.9993	0.9745	0.8131	0.8450	0.8096
daylight/EMS + SPI/BPI	0.9423	0.9994	0.9817	0.8462	0.8862	0.8259
daylight/EMS + SSI/BBI	0.9413	0.9994	0.9803	0.8167	0.8645	0.8070
SPI/BPI + SSI/BBI	0.8809	0.9985	0.9524	0.6384	0.7016	0.6135
daylight/EMS + SPI/BPI + SSI/BBI	0.9410	0.9994	0.9810	0.8399	0.9003	0.8208
CNN + daylight/EMS + SPI/BPI	0.9492	0.9996	0.9860	0.8461	0.8627	0.8506
CNN + daylight/EMS + SSI/BBI	0.9490	0.9996	0.9859	0.8582	0.8741	**0.8577**
CNN + SPI/BPI + SSI/BBI	0.9404	0.9994	0.9791	0.8120	0.8467	0.8086
CNN + daylight/EMS + SPI/BPI + SSI/BBI	**0.9676**	**0.9997**	**0.9892**	**0.8673**	**0.9039**	0.8509

The best performance is shown in bold

### PU-sampling evaluation

In related work, randomly selected instances from unlabeled data are used as negative DDI [7, 8]. This approach may introduce noisy data and lead to a lack of distinction between positive and negative samples. To test whether PU-sampling can accurately screen out high-confidence negative samples, we compare PU-sampling with traditional random sampling and the classical sampling method SMOTE [39]. As shown in Table 3, the results of traditional random sampling are significantly lower than the other two methods, proving the necessity of sampling negative samples in the DDI data set. In addition, PU-sampling outperforms SMOTE, verifying the effectiveness of PU-sampling in identifying noise in negative samples.

**Table 3:** The performance of Multi-SBI with random sampling and PU-sampling

Method	ACC	AUC	AUPR	F1	Pre	Rec
PU-sampling	**0.9676**	**0.9997**	**0.9892**	**0.8673**	**0.9039**	**0.8509**
SMOTE	0.9512	0.9994	0.9632	0.8456	0.8829	0.8398
random sampling	0.9101	0.9991	0.9586	0.8345	0.8693	0.8139

The best performance is shown in bold

### Comparison with existing state-of-the-art methods

**Fig. 6:**
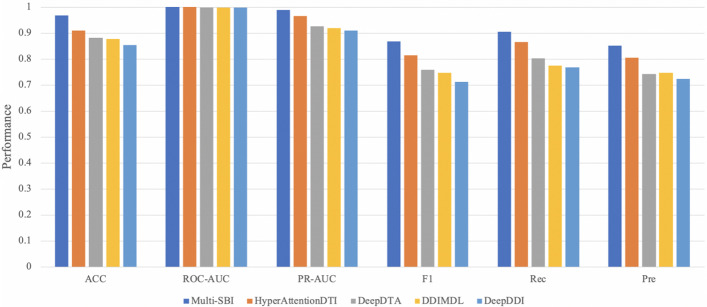
The performance of different methods

We compared Multi-SBI with the most advanced interaction prediction methods DDIMDL [8], DeepDDI [7], and drug-target prediction methods HyperAttentionDTI [18], DeepDTA [19]. Table 4 and Fig. 6 show the performance of Multi-SBI and the four methods on the test set. Because these baselines adopted the random-sampling strategy, Multi-SBI with different negative sampling would get another negative sample distribution. Thus, we added Multi-SBI (random-sampling) in Table 4 for a fair comparison. As we can see from the table, Multi-SBI (random-sampling) still led other advanced methods in five out of six metrics. It is found that all evaluation indicators obtained by Multi-SBI are higher than other methods. We can conclude that our method improves further with the enhancement of PU-sampling.

**Table 4:** The performance of different methods

Method	ACC	AUC	AUPR	F1	Pre	Rec
Multi-SBI	**0.9676**	**0.9997**	**0.9892**	**0.8673**	**0.9039**	**0.8509**
Multi-SBI(random-sampling)	0.9101	0.9991	0.9586	0.8345	0.8693	0.8139
HyperAttentionDTI	0.9093	0.9991	0.9652	0.8145	0.8653	0.8043
DeepDTA	0.8804	0.9983	0.9263	0.7594	0.8026	0.7628
DDIMDL	0.8764	0.9982	0.9196	0.7460	0.7934	0.7568
DeepDDI	0.8541	0.9979	0.9092	0.7129	0.7681	0.7230

The best performance is shown in bold

**Fig. 7:**
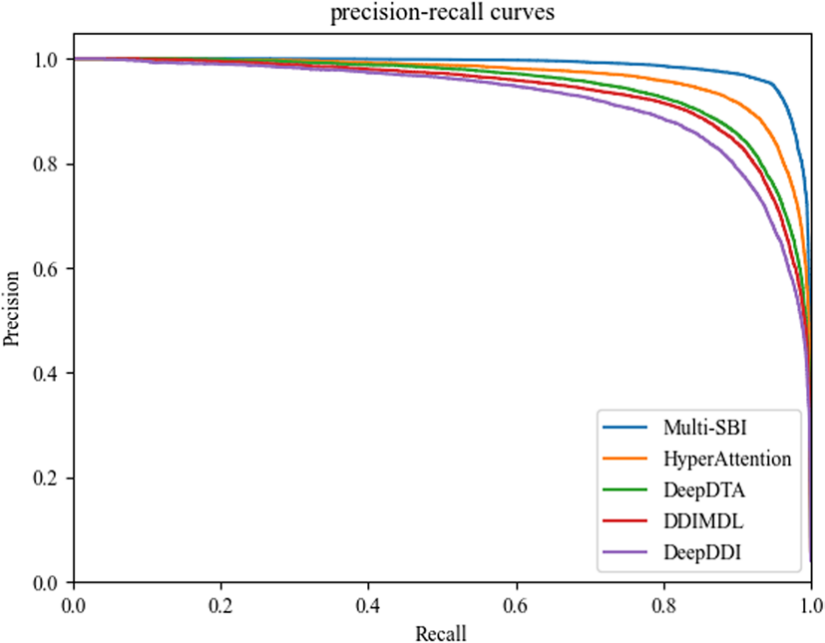
The precision-recall curves of different methods

In addition, the precision-recall curves of the above methods are shown in Fig. 7. We can see that the area under the precision-recall curves of Multi-SBI is more extensive than all other methods. These results go beyond previous reports, showing that Multi-SBI can effectively predict SBI.

During the experiments, we noticed that all the AUC metrics in different models were high (close to 1). So we analyzed the data distribution in Fig. 5. Most of the samples were concentrated in a few categories on the left side of the histogram (the first ten classes containing 90% data), which played a decisive role in the multi-classification tasks. Although the AUC metrics of the models were close to each other, our model performed well on the recall metric (Rec in Table 4) under both sampling mechanisms. The recall metric can reflect the ability to predict "Right" without considering the negative difference, which is acceptable to illustrate the capability of our model.

## Discussions

Very few computational methods can currently predict the interaction between SMDs and BioDs. Although determining the precise SBI is critical to improving patient care, it remains a challenging task that has not been fully studied through predictive modeling. This study proposes a multi-modal representation learning framework called Multi-SBI to predict potential SBI.

The feature representation of SMD and BioD drug pairs is much more complex than that of SMD drug pairs. We use multi-modal representation learning to represent drug pair features adequately. On the other hand, no specific database represents non-interacting drugs. We apply PU-sampling to filter unlabeled negative samples. The experiments demonstrate the ability of PU-sampling to remove imbalanced data set, and multi-modal features improve the performance of drug interaction prediction.

To fully demonstrate the ability of Multi-SBI to discover potential drug interactions, we perform retrospective analysis. In DrugBank 5.1.0, We obtained 8,547 drug interactions between 1,249 SMDs and 105 BioDs and used them as a training set for testing in unlabeled samples. The 14 out of the 20 drug pairs with the highest prediction scores can be found in the latest version of the DrugBank5.1.8, indicating the effectiveness of our model in predicting unknown drug interactions. The results are shown in Table 5.

**Table 5:** Top 20 prediction results from the retrospective analysis on DrugBank 5.1.0

No	SMD A	BioD B	*Event Type	Evidence
1	Glisoxepide	Nesiritide	1	N.A
2	Vorapaxar	Tocilizumab	2	DrugBank5.1.8
3	6-O-benzylguanine	Tocilizumab	2	DrugBank5.1.8
4	Raltitrexed	Protein S human	3	DrugBank5.1.8
5	Domoic Acid	Tocilizumab	2	N.A
6	Talazoparib	Tocilizumab	2	N.A
7	Glisoxepide	Insulin	3	DrugBank5.1.8
8	Glisoxepide	Insulin pork	3	DrugBank5.1.8
9	Vorapaxar	Siltuximab	1	DrugBank5.1.8
10	Domoic Acid	Siltuximab	1	N.A
11	Zolmitriptan	Tocilizumab	1	DrugBank5.1.8
12	Desonide	Tocilizumab	1	N.A
13	Glisoxepide	Insulin glulisine	3	DrugBank5.1.8
14	6-O-benzylguanine	Siltuximab	1	DrugBank5.1.8
15	Fluocinolone acetonide	Tocilizumab	1	DrugBank5.1.8
16	Fluocinonide	Tocilizumab	1	DrugBank5.1.8
17	Desonide	Siltuximab	1	N.A
18	Glisoxepide	Mecasermin	3	DrugBank5.1.8
19	Glisoxepide	Insulin detemir	3	DrugBank5.1.8
20	Glisoxepide	Insulin lispro	3	DrugBank5.1.8

*Event Type: 1: The metabolism of Drug A can be increased when combined with Drug B; 2: The risk or severity of adverse effects can be increased when Drug A is combined with Drug B; 3: The risk or severity of hypoglycemia can be increased when Drug A is combined with Drug B

## Conclusions

Identifying novel drug interactions is critical for improving clinical care. This paper presents a multi-modal representation learning method for interaction prediction between SMDs and BioDs. To our knowledge, this work is the first attempt to predict the interaction between SMDs and BioDs computationally.

On the one hand, in addition to the traditional method, we use two independent CNN-based blocks to extract the SMD and BioD sequences. On the other hand, we obtain the heterogeneous network information of the drug through one-hot encoding. Then, we use PU-sampling to obtain a balanced data set. Compared with previous methods of predicting drug interactions, Multi-SBI not only digs deep into the structural information of drugs but also considers node associations in heterogeneous networks. At the same time, the high-confidence negative sample set is selected. The prediction performance of our model in experiments has been significantly improved, and some new SBI predictions have been confirmed. These results show that Multi-SBI can provide a valuable tool for extracting and learning drug features to predict new SBI. It can provide biologists with SBI candidates, reduce the workload of wet laboratory experiments, and promote the development of new drug discovery and drug repositioning.

Despite the promising performance described above, our method still needs to address some limitations and provide insights for future research. First, the lengths of BioD sequences in the DrugBank database are pretty different. How to uniformly extract and characterize protein drugs of different lengths is still a complex problem, and we will improve this later. In addition, in the future, we will conduct biological experiments on the newly predicted drug pair to determine its authenticity.

## Data Availability

The datasets generated and/or analyzed during the current study are available in the DrugBank and Multi-SBI repository. https://go.drugbank.com/. https://github.com/marinehdk/Multi-SBI.

## Abbreviations

DDIs: Drug–drug interactions
SMD: Small molecule drugs
BioDs: Biotechnology drugs
PU-sampling: Positive-unlabeled sampling
DNN: Deep neural network
TIP: Three-picture information dissemination
CNN: Convolutional neural network
AUC: Area under the ROC curve
AUPR: Area under the precision-recall curve
SMILES: Simplified Molecular Input Line Entry System
CDK: Chemistry Development Kit
SPI: SMD-Protein Interaction
BPI: BioD-Protein Interaction
SSI: SMD-SMD interaction
BBI: BioD-BioD interaction
